# Inflammation Related MicroRNAs Are Modulated in Total Plasma and in Extracellular Vesicles from Rats with Chronic Ingestion of Sucrose

**DOI:** 10.1155/2016/2489479

**Published:** 2016-11-24

**Authors:** Malinalli Brianza-Padilla, Roxana Carbó, Julio C. Arana, Gonzalo Vázquez-Palacios, Martha A. Ballinas-Verdugo, Guillermo C. Cardoso-Saldaña, Adán G. Palacio, Yaneli Juárez-Vicuña, Fausto Sánchez, Eduardo Martínez-Martínez, Fengyang Huang, Fausto Sánchez-Muñoz, Rafael Bojalil

**Affiliations:** ^1^Posgrado en Biología Experimental, Universidad Autónoma Metropolitana-Iztapalapa, Av. San Rafael Atlixco No. 186, Col. Vicentina, Iztapalapa, 09340 Mexico City, Mexico; ^2^Departamento de Biomedicina Cardiovascular, Instituto Nacional de Cardiología “Ignacio Chávez”, Juan Badiano No. 1 Col. Sección XVI, Tlalpan, 14080 Mexico City, Mexico; ^3^Departamento de Inmunología, Instituto Nacional de Cardiología “Ignacio Chávez”, Juan Badiano No. 1 Col. Sección XVI, Tlalpan, 14080 Mexico City, Mexico; ^4^Colegio de Ciencias y Humanidades, Universidad Autónoma de la Ciudad de México-San Lorenzo Tezonco, Av. Prolongación San Isidro No. 151, Col. San Lorenzo Tezonco, Iztapalapa, 09790 Mexico City, Mexico; ^5^Departamento de Endocrinología, Instituto Nacional de Cardiología “Ignacio Chávez”, Juan Badiano No. 1 Col. Sección XVI, Tlalpan, 14080 Mexico City, Mexico; ^6^Ciencias Biológicas y de la Salud, Universidad Autónoma Metropolitana Xochimilco, Calzada del Hueso 1100, Villa Quietud, Coyoacan, 04960 Mexico City, Mexico; ^7^Instituto Nacional de Medicina Genómica, Periférico Sur No. 4809, Col. Arenal Tepepan, Tlalpan, 14610 Mexico City, Mexico; ^8^Laboratorio de Farmacología y Toxicología, Hospital Infantil de México Federico Gómez, Calle Dr. Márquez No. 162, Cuauhtemoc, Doctores, 06720 Ciudad de México, Mexico

## Abstract

Circulating microRNAs (miRNAs) and the functional implications of miRNAs contained in extracellular vesicles (EVs) have gained attention in the last decade. Little is known about the regulation of the abundance of plasma miRNAs in response to chronic ingestion of carbohydrates. Therefore, we explored the circulating levels of miR-21, miR-146a, miR-155, and miR-223 in rats consuming sucrose in drinking water. Weanling Wistar rats were 25 weeks with 30% sucrose in drinking water, and miRNAs expression was determined in total plasma and in microvesicles, by RT-qPCR with TaqMan probe based assays for miR-21, miR-146a, miR-155, and miR-223, using cel-miR-39 (as spike in control and reference). Endotoxemia was also measured. Sucrose-fed animals showed higher body weight and retroperitoneal adipose tissue as well as higher glucose and triglyceride plasma levels than controls. Plasma endotoxin levels were low and not different among groups. Plasma miR-21 and miR-223 were higher in the sucrose group (*p* < 0.05), whereas miR-155 tended to be lower (*p* = 0.0661), and miR-146a did not show significant differences. In the plasma EVs the same trend was found except for miR-146a that showed significantly higher levels (*p* < 0.05). Overall, our results show that high carbohydrate ingestion modulates circulating miRNAs levels related to an inflammatory response.

## 1. Introduction

Chronic ingestion of high amounts of carbohydrates contributes to the obesity epidemic worldwide [1]. Experimental models have been widely used to explore this phenomenon. It is known that high fructose and sucrose-fed animals reproduce the features of metabolic syndrome (MS) [2, 3]. Although many of the signals participating in the response of an organism to the continued exposure to high carbohydrate ingestion are reported in the literature, molecular signals through circulating noncoding RNA, such as microRNAs (miRNAs), are giving new insights.

miRNAs are small, noncoding RNA molecules of approximately 22 nucleotides in length that act as posttranscriptional regulators of gene expression [4]. In 2008, miRNAs were found in human serum and plasma [3, 5, 6] and are now useful biomarkers in many inflammatory conditions including obesity [7]. Some circulating miRNAs are considered molecular players of the innate immune response, especially if they are contained in extracellular vesicles (EVs) [8]. In this context, some circulating miRNAs can participate in inflammatory pathways. Such is the case of miR-21, miR-146a, miR-155, and miR-223 [9–11]. As has been demonstrated for inflammatory and immunity molecules, miRNAs expression can also be regulated by nutrients [12]. Many dietary compounds may modify miRNAs in cells and tissues; thus, circulating miRNAs levels can also be biomarkers of exposure to particular nutrients [13].

Increased consumption of simple carbohydrates such as fructose and sucrose has been linked to many pathophysiological processes and their effect on health is still controversial [14]. In particular, rats with chronic ingestion of sucrose after weaning in the drinking water as an unlimited beverage may display many signs of metabolic abnormalities such as moderate elevation of blood pressure, hypertriglyceridemia, hyperinsulinemia, excessive retroperitoneal fat and whole body fat [6], renal damage [15], and high vascular reactivity and disruption of innate inflammatory mediators [16]. Because all these metabolic disorders have been associated with inflammation we hypothesized that miRNAs involved in innate immunity (known as inflamma-miRs) may be altered in parallel to the metabolic disturbances induced by chronic ingestion of sucrose; thus, we aimed to evaluate the effect of chronic ingestion of sucrose on the levels of miR-21, miR-146a, miR-155, and miR-223, in total plasma and plasma extracellular vesicles of rats.

## 2. Materials and Methods

### 2.1. Animals

Fourteen weanling male Wistar rats weighing 70–95 g were randomly allocated into two groups. Control group was supplied with tap water ad libitum, whereas high sucrose drink group received a 30% sucrose solution in water, as their only liquid source. Animal feeding during 25 weeks consisted of a standard rodent diet (Laboratory Rodent Diet 5001: protein 28.507%, fat 13.496%, HCO 57.996%, from which sucrose 3.7%, fructose 0.3%, glucose 0.22%, PMI Nutrition International, Brentwood, MO). All animals were housed under artificial 12-hour light/dark cycles and a mean temperature of 22°C. The experiments in animals were approved by the Laboratory Animal Care Committee of our Institution and were in compliance with international ethical guidelines for animal research.

### 2.2. Serum Measurements

After 25 weeks, rats from both groups were weighed, fasted 12 h, and sacrificed. Blood samples were collected using K+EDTA as anticoagulant. Retroperitoneal adipose tissue was collected and weighed. Plasma was obtained by blood centrifugation (3000 rpm during 15 minutes at 4°C) and stored at –70°C until needed. Glucose was measured with a commercial enzymatic kit (DCL-glucose oxidase Diagnostic Chemical Limited de Mexico, Mexico). Insulin was determined with a commercial rat specific radioimmunoassay kit (Linco Research, Inc., Missouri, USA) with 0.1 ng/mL sensitivity and intra- and interassay coefficients of variation of 5 and 10%, respectively. Triglycerides and cholesterol were determined with commercially available procedures (Spinreact cholesterol-LQ and triglycerides-LQ; Spinreact S.A. Girona, Spain). HDL-cholesterol was measured by enzymatic procedures (Hitachi 902 analyzer; Hitachi LTD, Tokyo, Japan). Accuracy and precision of lipid measurements in our laboratory are under periodic surveillance as recommended by the Centers for Disease Control and Prevention (Atlanta, GA, USA). Plasma endotoxin levels were determined with GenScript Toxin Chromogenic LAL Endotoxin according to the manufacturer's instructions.

### 2.3. Plasma RNA Isolation

From the collected blood sample in K+EDTA, 100 *μ*L of plasma was processed for the isolation of RNA using the miRNeasy serum/plasma kit adding cel-miR-39 (1.6 × 10^8^ copies) spike in control (Qiagen) and 1 *μ*L of bacterial ribosomal RNA (Roche) according to the provider recommendation. Extracted RNA isolated from samples was stored at −70°C until processing.

### 2.4. Extracellular Vesicles RNA Isolation

500 *μ*L of plasma was processed for the isolation of RNA using the exoRNeasy serum/plasma midi kit. During the RNA purification step the same amount mentioned above of cel-miR-39 spike in control was added (QIagen) according to the provider recommendations and previous publication [17]. Extracted RNA isolated from EVs was immediately converted to cDNA as described below.

### 2.5. Determination of miRNAs by RT-qPCR

The miRNAs were detected and quantified using two-step RT-qPCR with RT-primer specific assay in combination with TaqMan probes (Applied Biosystems). Each RT-reaction used 1.5 *μ*L from the 14 *μ*L eluted RNA using the TaqMan MicroRNA Reverse Transcription Kit (Applied Biosystems). The RT-reaction program consisted of 30 minutes at 16°C, 30 minutes at 42°C, and 5 minutes at 85°C. The miRNAs were detected and quantified using miRNAs Assays hsa/mus/rno-miR-21, miR-146a, miR-155, and miR-223 primers and probes (Applied Biosystems). The 2 *μ*L of RT-reaction was amplified in 15 *μ*L reactions. PCR cycling conditions were initial denaturation at 95°C for 10 min, followed by 45 cycles at 95°C for 15 s, at 60°C for 60 s, and at 72°C for 1 s. PCR was performed using a LightCycler TM 480 II System (Roche Applied Science, Basel, Switzerland) with the LightCycler 480 Probes Master kit (Roche Applied Science). miRNAs relative concentrations were normalized with Ct values of cel-miR-39 and values were calculated using 2^−ΔΔCt^ and 2^−ΔCt^ formulas. All Ct values for cel-miR-39 ranged from 20 to 22 cycles both for total plasma and for EVs RNA isolations.

### 2.6. Particle Number Estimation

One mL of plasma was pipetted into a 1.5 mL tube and 400 *μ*L of PBS was added to each sample. The tubes were loaded into a fixed angle rotor (TLA 100.3; Beckman Coulter) for ultracentrifugation (Optima MAX Ultracentrifuge; Beckman Coulter) at 120,000 ×g at 4°C for 90 min. The pellets were resuspended in PBS and centrifuged again at 120,000 ×g for 90 min. The final pellet was resuspended in 50 *μ*L of PBS for nanoparticle tracking analysis (NTA). NanoSight NS300 was used to determine vesicle size and concentration (Malvern Instruments Ltd). Dilutions of 1 : 200 in PBS of each sample were injected into the NanoSight chamber. The camera gain was set at a constant value of 10 and the threshold value for vesicle detection was set at 5.

### 2.7. Statistical Analysis

Data are presented as means and standard errors. Data were tested for normality and equal variances. Accordingly, differences between groups were assessed by unpaired *t*-test or Mann-Whitney *U* test (*p* < 0.05) using the Graph Pad Prism software version 5.

## 3. Results

Rats in the high sucrose drink group had higher body weight and had almost three times more retroperitoneal fat than controls (*p* = 0.05 for both variables). Also, the sucrose-fed rats showed higher glucose levels and triglycerides than controls (*p* = 0.001). No differences between groups were observed for plasma insulin, endotoxins, and total HDL and LDL cholesterol (*p* > 0.05) (Table 1).

**Figure 1:**
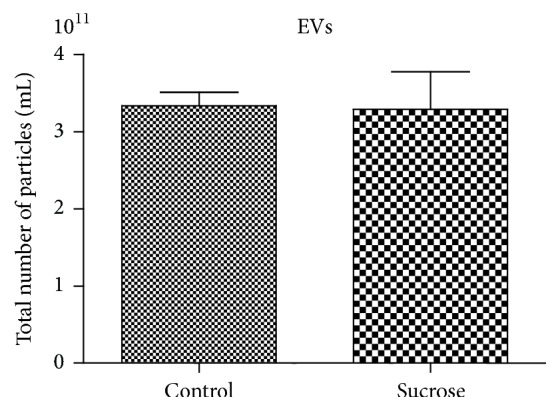
EVs assessed in plasma by particle number estimation in 3 control and 3 sucrose-fed rats. Means ± SE are shown and no differences were observed by Mann-Whitney *U* test (*p* > 0.05).

Previously in 3 controls and 3 sucrose-fed rats, we determined the amount of total extracellular vesicles and no differences between groups were observed. Since quantification of EVs is complex we supposed that the amount of EVs does not change with chronic sucrose ingestion as seen by total particle assessment (Figure 1).

**Figure 2:**
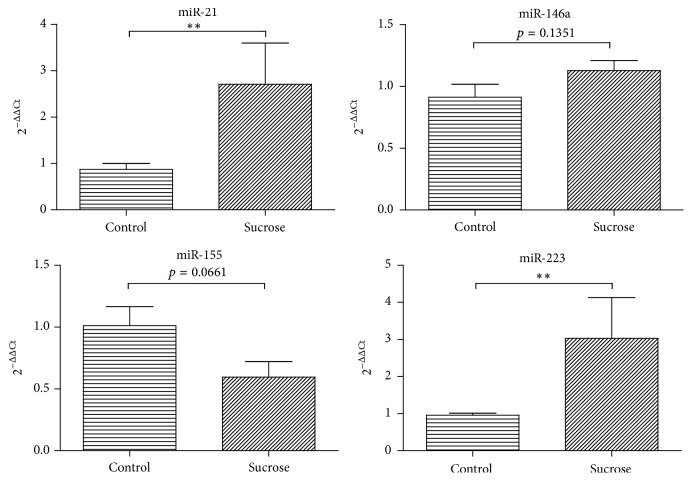
Plasma miRNAs levels in sucrose-fed rats (means ± SE). miR-21, miR-146a, miR-155, and miR-223 were measured in 7 animals per group by RT-qPCR using cel-miR-39 as a reference for the 2^−ΔΔCt^ method. Differences were tested by unpaired *t*-test or Mann-Whitney *U* test. ^*∗∗*^ *p* < 0.01.

The relative levels for the miR-21 and miR-223 were 2.7- and 3-fold higher, respectively, more abundant in the sucrose-fed animal groups when compared to the control group (*p* < 0.01). The plasma levels of miR-155 from the animals fed with sucrose had a nonsignificant tendency to be 40% downregulated when compared to the control group (*p* = 0.066). The levels of miR-146a were not different when compared to the control group (*p* > 0.05) (Figure 2).

**Figure 3:**
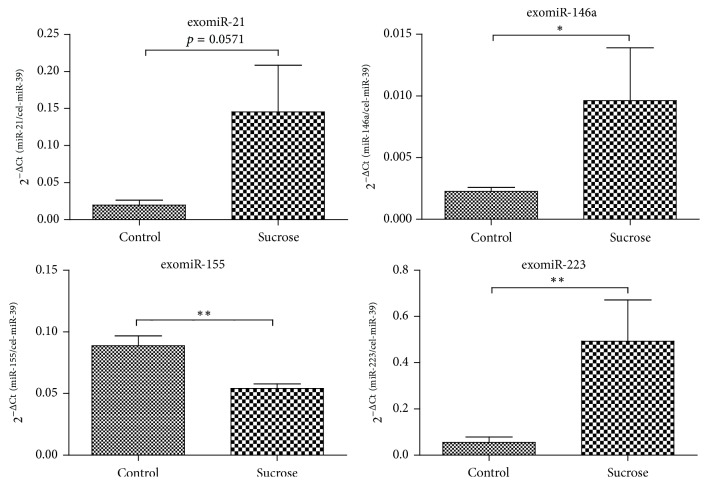
miRNAs levels in plasma extracellular vesicles of chronic sucrose-fed rats (means ± SE). RNA was isolated from plasma EVs, and the miR-21, miR-146a, miR-155, and miR-223 levels were measured in 4 animals per group by RT-qPCR using cel-miR-39 spike as a reference for the 2^−ΔCt^ method. Differences were tested by unpaired *t*-test or Mann-Whitney *U* test. ^*∗*^ *p* < 0.05, ^*∗∗*^ *p* < 0.01.

In plasma EVs the miRNA levels of miR-146a and miR-223 were found higher in the sucrose drink group as compared to the control group (*p* < 0.05 and *p* < 0.01, resp.). The miR-155 levels in the EVs had lower levels in the sucrose drink animals than in the control group (*p* < 0.05). For the miR-21 levels, only a trend for higher abundance was found in the sucrose group (*p* = 0.057) (Figure 3).

The relative abundance in total plasma as compared to the same amount of cel-miR-39 spike in control was miR-223 > miR21 > miR146a > miR-155. The relative abundance of miRNAs present in plasma EVs was miR-223 > miR-21 > miR-155 > miR-146a (Figure 3).

## 4. Discussion

In our study, chronic ingestion of sucrose induced changes in the concentrations of inflammation related miRNAs both in plasma and in plasma EVs. In agreement with previous findings in sucrose-fed rats by other groups [2] and by us [6], these rats had also higher body weight and visceral fat, as well as glucose and triglycerides levels. Insulin levels, total cholesterol, HDL, and LDL cholesterols were not found to be modified by sucrose. Because an endotoxemia secondary to changes in microbiota has been described in rats following a high fat diet [18], we measured plasmatic levels of endotoxin to assess if any changes of on miRNAs levels could be explained by this fact. No differences in endotoxemia were observed between groups, indicating that our findings may not be attributed to a similar phenomenon. Also, in a preliminary experiment we determined vesicle size and concentration in both rat groups, and the results were not different (*p* > 0.05). Thus, we assumed that EVs were not affected by chronic sucrose.

The changes observed in miR-21 total plasma and EVs, upon sucrose chronic exposure, are likely associated with the increased adipose tissue mass. Previous reports show that miR-21 levels increase in the white adipose tissue of mice with high fat diet-induced obesity and during human adipocyte stem cells proliferation [19]. Also, upregulated miR-21 levels in serum are associated with nonalcoholic fatty liver disease, especially in men [20]. Accordingly, 20% consumption of sucrose has been reported associated with mild liver steatosis in rats [21]. This miRNA may have a role in sustaining adipose tissue expansion as reported in a study using miR-21 antagomiRs in the db/db mice [22].

The higher levels of miR-146a observed only in the RNA from the plasma EVs in the sucrose group may consider that miR-146a levels are associated with several diseases, including diabetes [23, 24]. Since in our experiment the sucrose group rats had a mild hyperglycemia, we think that, as others have suggested, miR-146a upregulation through EVs may be an anti-inflammatory mechanism important in the controls of insulin sensitivity induced by inflammatory mediators [25]. Thus, it is possible that upregulation of circulating miR-146a on hyperglycemia may start in EVs, as seen in our chronically exposed rats. In patients with newly diagnosed type 2 diabetes miR-146a is elevated [24] and may diminish as disease progresses [23]. We also found lower levels of miR-155 in plasma EVs, correlated with total plasma levels. This reduction may be explained by the expansion of the adipose tissue found in our sucrose group of rats. Accordingly Chen and collaborators showed that miR-155 and C/EBP*β* constitute a bistable system for the regulation of adipogenesis [26]. In inflammation, evidence so far presented on miR-155 function indicates that it is likely to be pro- rather than anti-inflammatory [27]. Although, it has been recently reported by Li and collaborators that miR-155 is overexpressed in the plasma from patients with atherosclerosis and may have a key role in the anti-inflammation activity of macrophages, attenuating foam cell formation [28].

The changes seen in the expression of miR-146a and miR-155 may reflect part of the functional adaptations after a chronic exposure to high sucrose, in this case probably related to the innate immune response. In a model of endotoxemia in mice, it has been reported that exosomal miR-146a inhibits while miR-155 promotes the inflammatory response in some contexts [29]. Thus, the alternated increase of miR-146a and reduction miR-155 in plasma EVs could be part of the miRNA-mediated modulation of the inflammatory response.

We found miR-223 upregulated in both plasma and plasma EVs from the sucrose group of rats. These results are opposed to others previously reported in obese [30, 31] and type 2 diabetic individuals [32], in whom downregulation of miR-223 was found. Another study, however, found that levels were unchanged in diabetic subjects [33]. Previous studies using also chronic ingestion of sucrose found high levels of adiponectin [6, 16]. In the adipose tissue miR-223 suppresses proinflammatory activation of macrophages [34] and probably contributes to the results showing high levels of adiponectin in sucrose ingestion [6]. Also, this upregulation of miR-223 may in part account for the unchanged levels of circulating IL-1*β* in six months and its downregulation after 12 months [16]. It has been recognized that miR-223 negatively regulates NLRP3 and therefore IL-1*β* production [35].

Our results suggest that high sucrose consumption may induce a low grade inflammatory state characterized by a decrease in miR-155 with the increase of miR-21, miR-146a, and miR-223 in EVs. The results presented herein gain relevance in light of recent evidence showing that a horizontal vesicle-mediated transfer of miRNAs allows the intercellular dissemination of gene expression regulatory messages, which may modify the function of target cells. Interestingly, exosome produced by macrophages upon administration to mice migrate into the adipose tissue [36]. Further studies are needed to clarify the cells originating the changes in EVs miRNA composition upon chronic consumption of sucrose.

## 5. Conclusions

Chronic ingestion of sucrose induced the upregulation of miR-21 and miR-223 in plasma and EVs. Interestingly, the combined upregulation of miR-21 and downregulation of mR-155 may possibly be responsible of high carb diets (in this case sucrose) mediating the adipose tissue expansion. Thus, we hypothesize that inflammatory modulation triggered by the high availability of simple carbohydrates from early life may force the organism to seek homoeostatic mechanisms including regulation by inflamma-miRs.

## Figures and Tables

**Table 1:** Body weight central adiposity and biochemical means (±SE) related to metabolic syndrome.

	Control	Sucrose drink	*p* value^*∗*^
Weight (g)	460 ± 18.4	565 ± 27.4	0.05
Blood pressure (mmHg)	124 ± 5.6	132.3 ± 10.5	n.s.
Retroperitoneal fat deposits (g)	5.25 ± 0.8	14.02 ± 2.4	0.05
Glucose (mg/dL)	87.7 ± 8.6	105 ± 6.2	0.05
Triglycerides (mg/dL)	58.5 ± 12.7	117.8 ± 17.3	0.001
Cholesterol (mg/dL)	51.2 ± 4.9	52.9 ± 4.1	n.s.
HDL-cholesterol (mg/dL)	39.4 ± 3.7	36.0 ± 1.8	n.s.
LDL-cholesterol (mg/dL)	6.2 ± 0.9	7 ± 1.3	n.s.
Insulin (*µ*UI/mL)	11.5 ± 2.3	12.0 ± 2.3	n.s.
Endotoxin (EU/mL)	0.0276 ± 0.0048	0.0332 ± 0.0088	n.s.

^*∗*^Means were separated by unpaired *t*-test or Mann-Whitney *U* test.
